# Design of Spectrum Processing Chiplet Based on FFT Algorithm

**DOI:** 10.3390/mi14020402

**Published:** 2023-02-07

**Authors:** Baoping Meng, Guangbao Shan, Yanwen Zheng

**Affiliations:** School of Microelectronics, Xidian University, Xi’an 710071, China

**Keywords:** electromagnetic spectrum, spectrum processing, chiplet, FFT

## Abstract

With the rapid development of electronic information and computer science, the fast Fourier transform (FFT) has played an increasingly important role in digital signal processing (DSP). This paper presented a spectrum processing chiplet design method to solve slow speed, low precision, and low resource utilization in spectrum processing of general-purpose spectrum chips and field programmable gate array (FPGA). To realize signal processing, the Radix-2 4096-point FFT algorithm with pipeline structure is used to process spectral signals extracted from the time domain. To reduce the harm caused by spectrum leakage, a windowing module is added to optimize the input data, and the clock gating unit (CGU) is used to perform low-power management on the entire clock reset. The result shows the chiplet takes 0.368 ms to complete a 4096-point frequency sweep under a clock frequency of 61.44 MHz. The chiplet significantly improves speed and accuracy in spectrum processing, which has great application potential in wireless communication.

## 1. Introduction

As a carrier of information, electromagnetic spectrum equipment has great application value in wireless communication, aerospace, radar, GPS, etc. [1]. There are higher requirements for the real-time and accuracy of electronic equipment information processing with the increasing complexity of the electromagnetic environment [2]. Thus, it is critical to design a processor with real-time and accurate processing of electromagnetic spectrum signals. Moreover, as electronic devices continue to get smaller, there is urgent need to further reduce redundant hardware resources and lower power consumption.

In order to achieve the real-time, miniaturization, and low-power requirements of electromagnetic spectrum processing, a lot of attempts have been made by scholars and engineers to optimize the algorithm and hardware implementation of FFT. Currently, the main hardware implementations of FFT are general-purpose computer, digital signal processing (DSP), field programmable gate array (FPGA), and application-specific integrated circuit (ASIC). Relying on a general-purpose computer and the corresponding software to implement the FFT is generally used for the preliminary study of FFT algorithms, which is very easy to implement and can be modified at any time. However, the computational speed is so limited that the signal cannot be processed in time, and it is not suitable for FFT algorithms with a large number of points. DSP has very high universality and is also applicable to FFT computation for a wide range of application scenarios [3]. Jijin Xia et al. implemented an 8-channel parallel FFT transform process based on a multicore DSP, which improved the processing performance with a computation time of 983.75 μs for a 32k-point FFT [4]. However, the generality of the DSP also leads to its drawbacks, as it has a large amount of redundant computational logic. Moreover, the parallelism of DSP depends on the number of its cores, so it cannot meet the demand in high-speed scenarios. FPGA, on the other hand, has a much higher degree of parallelism than DSP and can use rich logic resources through a parallel flow design approach to obtain a great increase in processing speed with less power consumption, which is more suitable for real-time processing of high-volume streaming data in embedded application scenarios. FPGA also has a configurable hardware structure, which makes them more flexible in pre-design and verification [5]. Therefore, the implementation of large point FFT using FPGA is a more enthusiastic way for researchers in the field of high-performance signal processing or high-speed computing. Aditya Sankaran et al. used Xilinx Virtex-7 XC7VX330T to implement a 4-channel parallel FFT computational structure based on the Radix-4 FFT algorithm to achieve real-time processing of FFT with 1k point FFT computation time of 220 μs [6]. Yongrui Li et al. chose the xc7vx690tffg1761-2 board under the Virtex7 series as the hardware platform with a 4-channel parallel, 4-stage pipelined computation with 128k-point FFT computation requiring 131,218 clock cycles [7]. Compared to FPGA, ASIC enables full customization and guarantees the reliability, real-time, and low-power requirements of the design. ASIC not only has a relatively low cost per chip, but also makes the design and flow of it much cheaper due to the mass production of ICs [8]. There are not only commercialized FFT chips dedicated to spectrum processing, such as the PDSP16570 from Plessy, but also self-developed and designed ASICs. For example, Samudrala H K, Qadeer S et al. implemented a 64-point FFT chip with a parallel pipeline architecture based on rotation factor scaling technique to reduce the complexity of the FFT algorithm. Compared with the traditional architecture, it reduces the hardware area by 13.74% and power consumption by 16% [9].

As the semiconductor industry enters the post-Moore era, the chiplet has received widespread attention due to its high performance, low power consumption, high area utilization, low cost, and reusability, and is considered as one of the key development directions for future integrated circuits [10,11]. Therefore, we propose a chiplet-based implementation of the FFT algorithm, whose implementation process is essentially similar to that of an ASIC, without the need to pursue extreme feature sizes while ensuring proper functionality.

In this paper, the chiplet based on FFT algorithm is designed for efficient and high-precision processing of spectrum processing signals, which expands on our previous work [12]. The pipeline structure and windowing module are designed to improve the speed and accuracy of hardware calculations, respectively. Furthermore, the clock gating unit (CGU) is used to perform low-power management on the entire clock reset. The overall design realizes the conversion of spectrum data from the time domain to the frequency domain, achieves the goal of high speed and accuracy, and verifies the broad application prospect of chiplet in electromagnetic spectrum processing.

## 2. System Architecture

### 2.1. FFT Introduction and Implementation

The chiplet design of spectrum processing module is based on discrete Fourier transform [13]. According to the design requirements, the 4096-point Radix-2 FFT algorithm with cascade structure is used to achieve the customized chip.

Defining a time series *x*(*n*) with length *N*, its DFT transformation formula is represented as (1):

(1) $$X(k)=\mathrm{DFT}[x(n)]=\sum_{n=0}^{N-1}x(n)W_{N}^{nk},\;0\leq k\leq N-1$$

where $W_{N}^{nk}$ is the rotation factor and *k* can be 0, 1, …, *N* – 1 [14]. According to the formula above, the DFT calculation of discrete sequence *x*(*n*) at *N* points is to obtain the corresponding value of *X*(*k*) in the frequency domain by summing the product of sequence *x*(*n*) and rotation factor $W_{N}^{nk}$. FFT is to continuously decompose the DFT of long sequence operators into the short sequence operators according to the periodicity and symmetry of rotation factor *W*, so as to reduce the amount of computation at each stage [15].

According to the parity of serial number *n*, the time-domain discrete sequence is divided into two sequences *x*_1_(*l*) and *x*_2_(*l*) of both length *N*/2, and then the *N*-point DFT can be decomposed into two *N*/2-point DFT to calculate. *X*_1_(*k*) and *X*_2_(*k*) are, respectively, used to represent the DFT operation results of *x*_1_(*l*) and *x*_2_(*l*) at *N*/2 points. Based on the symmetry of the rotation factor $W_{N}^{nk}$ and the hidden periodicity of *X*_1_(*k*) and *X*_2_(*k*), the following Equation (2) can be obtained:

(2) $$\begin{array}{l} X(k)=X_{1}(k)+W_{N}^{k}X_{2}(k)\\ X\left(k+\frac{N}{2}\right)=X_{1}(k)-W_{N}^{k}X_{2}(k) \end{array}\}\;k=0,1,\cdots \frac{N}{2}-1$$

**Figure 1:**
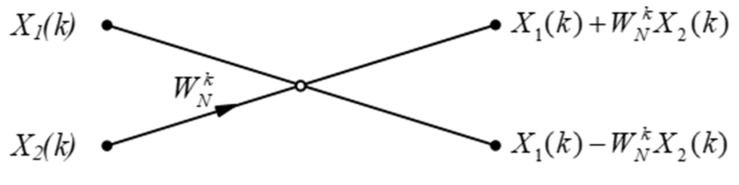
Butterfly element.

The butterfly element corresponding to (2) is shown in Figure 1 [16].

**Figure 2:**
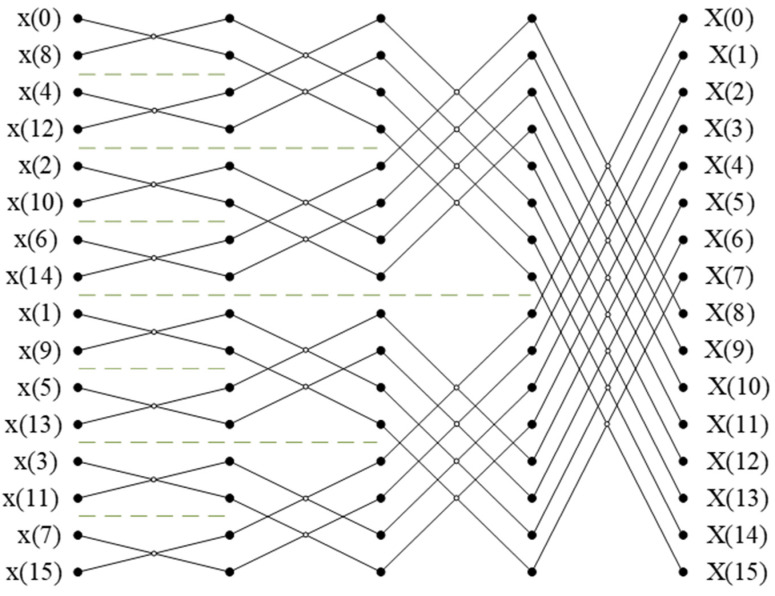
Calculation flow diagram of FFT.

In this way, an *N*-point DFT can be decomposed into smaller operation modules. After m-stage time-domain decomposition for odd and even sequences, it can be decomposed into *N* DFTs of 1-point and m-stage butterfly operations, which is FFT operation. According to the time-domain extraction algorithm, the data are arranged first, the decomposition is carried out in reverse order, and finally, the sequential output data are obtained. When *N* = 16, the computation flow diagram of FFT is shown in Figure 2.

The spectral data operation of *N*, which equals 2*^m^*, can be divided into *m* stages. Each stage has *N*/2 butterfly operation units, and a butterfly operation consists of two complex additions and one complex multiplication, so the *m* stages have *N*log_2_*N* addition units, *N*/2log_2_*N* multiplication unit, and the data are decomposed and processed by FFT operation, which significantly reduces the complexity of the operation.

**Figure 3:**
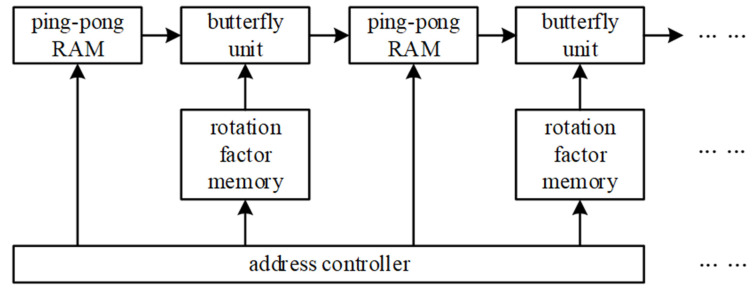
Architecture diagram of the cascade structure.

The architecture diagram of the FFT is shown in Figure 3. In the cascade structure, the ping-pong RAM structure is used to store data and send real-time data to the butterfly unit behind. By controlling the address, the rotation factor in the memory is given to the butterfly unit. The first butterfly unit completes the first base-determining calculation, the second butterfly unit completes the second one, etc. Since the number of butterfly operation units in the cascade structure is large enough, and it is convenient to adopt the pipeline design method in the calculation structure, the use of a cascade structure enables higher speed.

### 2.2. Hardware Division and Low-Power Design

**Figure 4:**
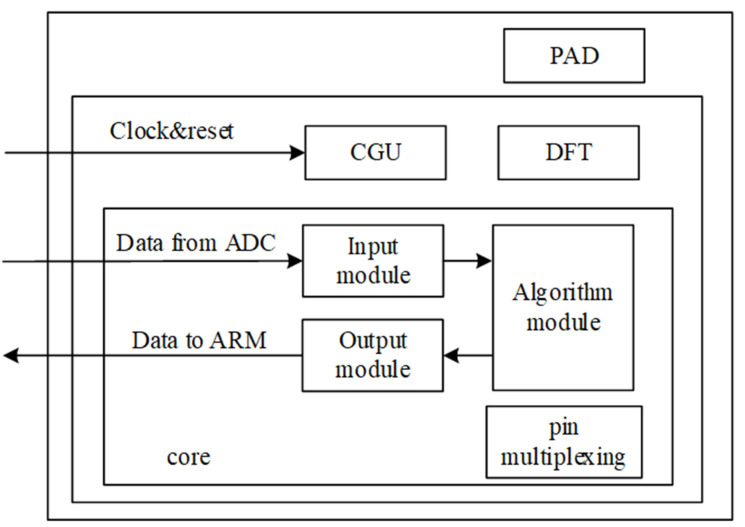
Hardware level of the spectrum processing module.

The design frame circuit of the spectrum processing module consists of three levels, as shown in Figure 4. The core layer of the top-level design is composed of the core calculation module, the second layer is composed of the clock gating unit (CGU) module and the design for testability (DFT) module, and the top layer is composed of the PAD planner. The core includes the algorithm module, input and output module, and pin multiplexing module. The CGU module manages the system clock and reset and reduces the inversion of the clock signal of each module. In this paper, in order to ensure the reliability of the circuit design of the spectrum processing module, the DFT test module is designed and implemented by means of pin multiplexing and data selectors. The DFT module consists of two parts, namely the insertion of the scan chain and the test design of the internal memory.

In the chiplet design process, the power consumption is partly caused by the distribution network of clock and reset. If a system has many clock matching units and buffer units, additional drivers are required to reduce the influence of clock delay. These matching units and buffer units also bring out the problem of dynamic power consumption. In addition, the power consumption caused by the inversion of the clock signals cannot be underestimated. Therefore, when the chiplet design of the spectrum module is performed, the clock and reset management of the entire design system is required.

**Figure 5:**
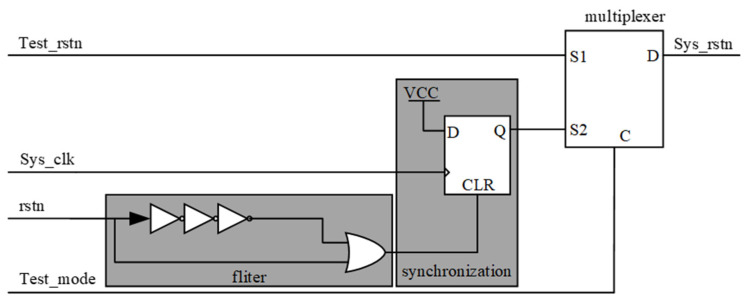
The reset structure.

The reset structure is shown in Figure 5. System reset is achieved through filter circuits, synchronization modules, and multiplexers. This is because there will be a certain probability of glitches when the signal jumps, which needs to be processed by the filtering and synchronization modules. Furthermore, the advancement of the cycle will lead to the update of the sampled data, and the stored value in the DFF will change, so a multiplexer is added to maintain the previous value and achieve the function of storage. The test_rstn signal is a DFT interface signal and is only valid when the test mode is selected. The system clock sys_clk acts on the global signal, AD9361 interface, and readout interface.

## 3. Critical Module Design

**Figure 6:**
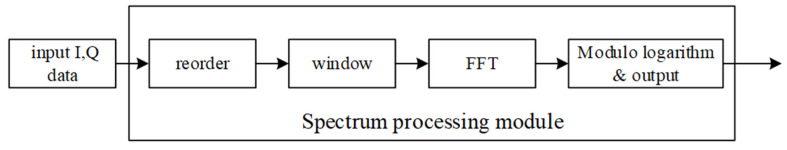
Algorithm implementation of the spectrum processing module.

Based on the FFT principle described in Section 2, the hardware of spectrum processing is designed. The spectrum processing module receives the 12-bit wide I and Q digital data output by the AD9361 radio frequency module. For the clock mismatch between the real and quantity signals of the serial output of AD9361, it is necessary to design an input control module to match the data and complete the 61.44 MHz cross-clock synchronization. Before FFT processing, it is generally necessary to truncate the original infinitely long signal, while the signal distortion is caused by the non-periodic nature of ADC sampling. This result is called spectrum leakage. To reduce the influence caused by spectrum leakage on the calculation, a windowing design needs to be performed on the reordered data. The spectrum processing reorders the data according to the pipeline-based time extraction algorithm to achieve the effect of inputting data in reverse order and outputting data in sequence. Finally, the logarithmic modulus operation is performed to realize the data output of the spectrum processing module. The algorithm implementation of the spectrum processing module is shown in Figure 6.

### 3.1. Reordering Module Design

According to the butterfly unit operation rules of the FFT algorithm, when the cascade structure is used for processing, the input and output data of the same butterfly unit are stored in the same memory, and this calculation is called co-location operation. Then, the relevant points of the entire pipeline computing module are correlated with co-location operation to realize co-location processing of 4096-point input data and intermediate data. In order to realize co-location operation and achieve the effect of out-of-order input and sequential output of spectral data, the position of the collected discrete time-domain signals is changed, and the final arranged out-of-order data are input to the calculation module by using the decomposition rule.

### 3.2. Data Windowing Module Design

When processing the original signal, due to the restriction of the hardware device, it cannot process the data of infinite length, so it is necessary to truncate the original data. This truncation mechanism will lead to leakage errors. At the same time, when the analog–digital converter (ADC) performs signal sampling, the sampled signal is often aperiodic, which causes the spectrum obtained at the sampling points to be distorted, and the spectrum energy is dispersed into a wider frequency band, causing spectrum leakage. In these two cases, an appropriate window function is selected to reduce the leakage error in the frequency domain due to signal truncation and signal sampling.

The expressions of different window functions are also different, and the cosine window function is widely used in spectrum processing. Cosine windows include rectangular windows, Hamming windows, Hann windows, Blackman windows, etc. The spectral characteristics of each cosine window are shown in Table 1 [17].

There are three influencing factors of the window function: the main-lobe width, the maximum side-lobe level, and fall-off of the side-lobe. It can be seen from the above table that the main-lobe width value of the Hann window function is moderate, so the resolution is high, the maximum side-lobe level is small, and the drop rate of side lobe reaches 18 dB/oct, resulting in less spectrum leaked, meeting the requirements of this project design. After comprehensive consideration, the window function selected in this project is the Hann window.

**Figure 7:**
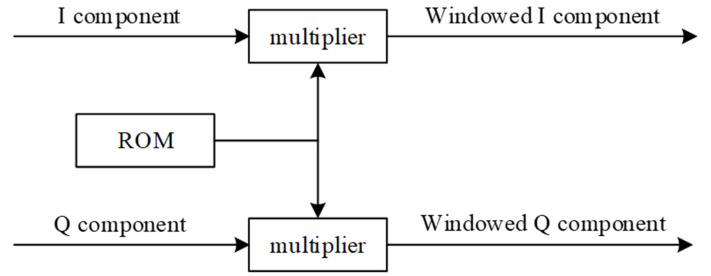
Windowing module.

According to the design, use MATLAB to obtain the data of the corresponding Hann window function, store the data in the ROM, and use the multiplier to perform window processing on the spectral signals I and Q. The windowing module is shown in Figure 7.

### 3.3. FFT Module Design

The calculation module based on FFT is the main algorithm module of spectrum transformation. According to the design idea proposed above, it realizes the FFT calculation of 4096 points, Radix-2, and cascade structure to process spectral signals extracted from the time domain. In the FFT data calculation of Radix-2, it is necessary to solve the product problem of discrete sequence *x*(*n*) and rotation factor. Both discrete sequence and rotation factor are plural numbers. The multiplication of plural numbers is much more complex than the multiplication of real numbers. Generally, the plural multiplication is represented by Equation (3):

(3) (a+bi)×(c+di)=(a×c−b×d)+i(a×d+b×c)

It can be seen from the equation above that the multiplication of two plural numbers is realized by four multiplications of real numbers. Still, in hardware design, the multiplier consumes more resources than the adder, which is one of the central units affecting power consumption and area. One way to modify (3) is to remove a multiplication operation at the cost of increasing the number of additions from 3 to 7. In chiplet design, the implementation resources of addition are much smaller than the resources of multiplication. This optimization significantly reduces the resource consumption of hardware, which is expressed by (4):

(4) $$\begin{array}{l} m_{1}=(a+b)\times c\\ m_{2}=(d-c)\times a\\ m_{3}=(c+d)\times b\\ \left(a+bi)\times (c+di)=(m_{1}-m_{3})+i(m_{1}+m_{2}\right) \end{array}$$

For the 4096-point FFT calculation, the Radix-2 algorithm requires a total of 12 stages of computing unit operations. The pipeline structure is used to cascade multi-stage Radix-2 processing. Each stage is configured with a Radix-2 computing unit, which requires a total of 12 stages and a maximum of 2048 iterations. In the case of multi-stage calculation, the pipeline structure is used for processing and each stage unit completes the operation independently and is stored separately, which significantly reduces the time of frequency spectrum processing.

**Figure 8:**
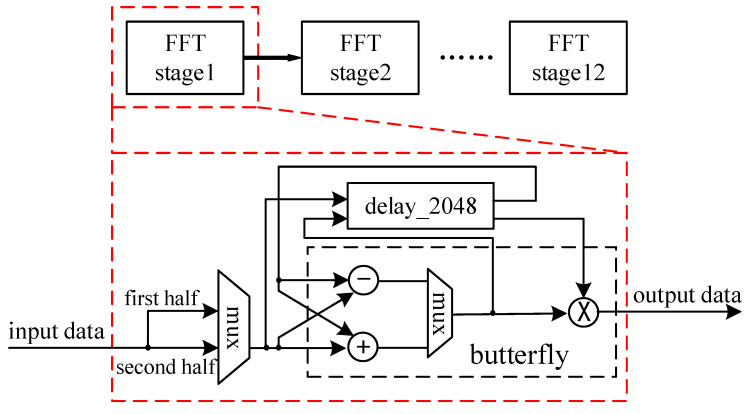
Pipeline structure of FFT.

The entire FFT hardware structure is shown in Figure 8. The main structure of the FFT algorithm of the spectrum module is a pipeline structure. The FFT calculation module includes calculation units FFT stage1, FFT stage2…FFT stage12. Each stage of the calculation unit includes a butterfly operation unit, a selection unit, a buffer unit, and a shift register. The FFT calculation module has the following characteristics:

- It consists of 12 cascaded stages; each stage contains a Radix-2 butterfly computing unit;
- Each stage has a set of FIFOs. The length of the FIFO is determined by the location of the stage. The depth is selected according to the time-domain extracted signals; the circular buffer unit and the shift register are combined to achieve different series of butterfly operations;
- Due to the use of cascade structure, the FFT calculation module can obtain very strong throughput;
- The FFT calculation module uses the selector to select data between the FIFO and the butterfly unit.

As shown in the figure above, each stage of FFT includes a delay unit. Its delay depth is:

(5) $$D=2^{P},\;P_{\max}=M$$

Among them, *D* represents the depth of the delay unit. *M* is the number of stages of the spectrum processing module. In this project, its value is 12, and *P* represents the number of stages of the current operation. As shown in Figure 2 and Figure 8, taking the first FFT unit as an example, the input data are controlled by the first data selector. After the first data enter the delay unit and wait for one clock cycle, the addition and subtraction operations are performed synchronously with the last input data. Under the control of the second data selector, the addition calculation result is multiplied by the rotation factor and then output to the next stage. The subtraction calculation result is delayed by one clock cycle and then multiplied by the rotation factor to output to the next stage, thus completing the FFT calculation of the first stage. In the same way, the next-stage FFT calculation is continued, and the entire FFT calculation realizes data pipeline processing by means of feedback delay.

### 3.4. Plural Modulo and Logarithmic Computation Module Design

The FFT calculation of Radix-2 realizes the conversion from the time domain to the frequency domain. Since the spectral data are usually in dB, in order to obtain a more standard spectral data format, the final output of the spectral module data is realized through the plural modulo and logarithmic computation module. After passing through the FFT calculation module, i.e., the core module, the spectral data are stored as the real part and the imaginary part, respectively. The final spectrogram is represented by the amplitude value of the spectral data; so, it is necessary to perform the modulo calculation on the calculated spectral data and obtain the amplitude value of the spectral data by taking the square root of the sum of the squares of the real and imaginary signal values.

Then, logarithm calculation is carried out. There are two methods for logarithmic computation: one is the coordinate rotation digital computer (CORDIC), and the other is the lookup table (LUT). This paper uses the CORDIC, which actually uses the data recursion method to simplify complex logarithmic operations into shift and addition and subtraction operations through the idea of approximation.

## 4. Analysis of Simulation Results

In the functional simulation, the signal source Rohde&Schwarz SMBV100B is used to generate the signal spectrum. Since the operating frequency limit of the AD frequency module is as high as 6 GHz, in order to verify the processing capability of the spectrum processing module, the high frequency signal is selected as input spectrum for processing. The three single-frequency signals of 4780 MHz, 4820 MHz, and 4900 MHz are selected for testing to ensure that the simulation results are more convincing.

**Figure 9:**
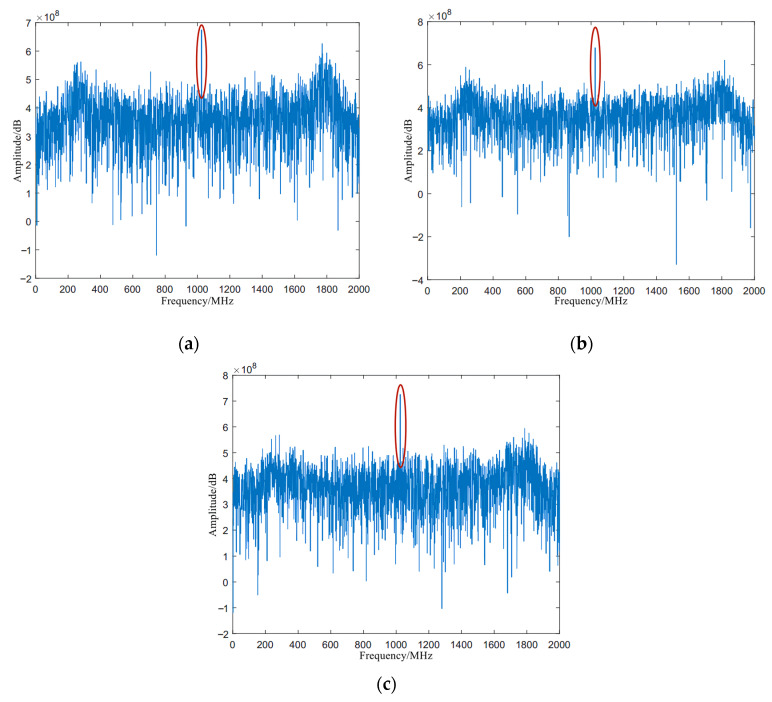
Spectrum processing diagram at three different frequencies: (**a**) spectrogram when the frequency of the input signal is 4780 MHz; (**b**) spectrogram when the frequency of the input signal is 4820 MHz; and (**c**) spectrogram when the frequency of the input signal is 4900 MHz.

Based on the above design approach and the verification idea, we finally selected the ZYNQ 7035 processor of Xilinx as the verification platform. The test results at three different frequencies are shown in Figure 9.

According to the above test method, the frequency of the input signals are 4780 MHz, 4820 MHz, and 4900 MHz, respectively, corresponding to (a), (b), and (c) of Figure 9. Additionally, the results of spectral processing are also clearly visible. Since the spectrum of the signal after processing of Fourier transform is symmetrical about the y-axis, half of the calculation points of the spectrum module is selected for display. The results show that for three input signal sources with different frequencies, the signal spectrum lines are successfully displayed at the center of the figure, and the rest is the background noise. So far, the spectrum processing function has been realized.

The calculation time is an intuitive indicator of the real-time processing performance of the FFT, and the number of clock cycles required for the FFT calculation can be obtained from the simulation results in the Vivado. After testing, it takes 0.368 ms to calculate a set of 4096-point data under the 61.44 MHz clock.

Based on the preliminary research work, we selected the National Institute of Technology Tiruchirappalli’s FPGA-based FFT implementation as a comparison [6], as both designs have similar algorithm models and verification platforms. The specific comparison results are shown in Table 2. It is clear that this work takes only 67.3% more time than the control group at 4 times the number of points and almost half the operating frequency.

**Figure 10:**
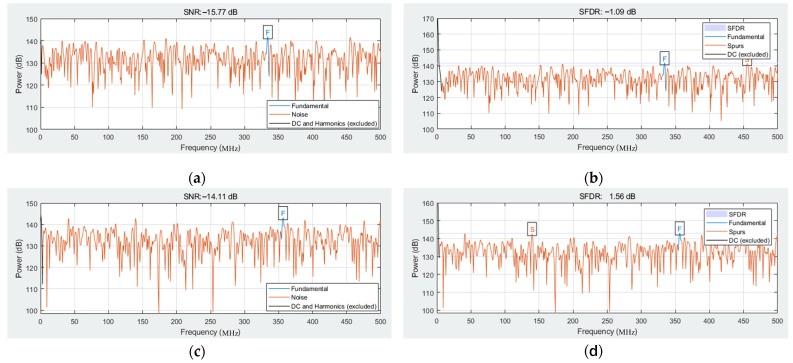

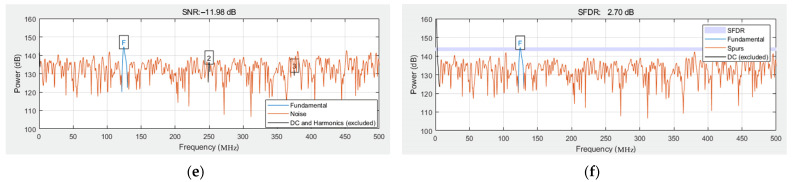
Extracted SNR and SFDR parameters: (**a**) extracted SNR when the frequency of the input signal is 4780 MHz; (**b**) extracted SNR when the frequency of the input signal is 4780 MHz; (**c**) extracted SNR when the frequency of the input signal is 4820 MHz; (**d**) extracted SNR when the frequency of the input signal is 4820 MHz; (**e**) extracted SNR when the frequency of the input signal is 4900 MHz; and (**f**) extracted SNR when the frequency of the input signal is 4900 MHz.

Then, the parameters of signal to noise ratio (SNR) and spurious-free dynamic range (SFDR) are extracted and analyzed. Table 3 and Figure 10 show the SNR and SFDR of the three input signal sources. Due to a large amount of noise in the input signal, the obtained SNR and SFDR values are relatively small, but the spectrum of the displayed signal can be accurately captured in the spectrogram. At the same time, it also shows that the spectrum processing module designed this time can perform high-precision operations in a complex electromagnetic environment to meet the high-precision design requirements.

## 5. Conclusions

This paper presents the chiplet-based hardware design to process spectrum processing signals efficiently and accurately. Specifically, a pipeline-based high-throughput, high-accuracy, 4096-point, spectral processing architecture is selected and designed. It has been verified that the spectrum processing function of chiplet design can be realized typically in a complex spectrum environment, and the time to calculate a set of 4096-point data under the clock of 61.44 MHz is 0.368 ms, which fully utilizes the advantages of large-point and high-speed computing of chiplet. The research proves that the application of chiplet technology to spectrum processing will have great prospects.

## Figures and Tables

**Table 1:** Comparison of spectral characteristics of different cosine windows.

Type of Window	Window Length	Main-Lobe Width	Maximum Side-Lobe Level/(dB)	Side-Lobe Fall-Off/(dB/oct)
Rectangle window	N	4π/N	−13	6
Hann window	N	8π/N	−31	18
Hamming window	N	8π/N	−43	6
Blackman window	N	12π/N	−58	18

**Table 2:** Comparison of simulation results.

Parameter	[6]	This Work
Verification Platform	Virtex-7	ZYNQ 7035
FFT Size	1024	4096
Algorithm	Radix-4	Radix-2
Operating Frequency	117 MHz	61.44 MHz
Execution Time	0.22 ms	0.368 ms

**Table 3:** Parameters of SNR and SFDR.

Frequency (MHz)	SNR (dB)	SFDR (dB)
4780	−15.8	−1.0
4820	−14.1	1.56
4900	−11.98	2.70
